# Targeting Inflammatory-Mitochondrial Response in Major Depression: Current Evidence and Further Challenges

**DOI:** 10.1155/2020/2972968

**Published:** 2020-04-14

**Authors:** Ana Paula Vargas Visentin, Rafael Colombo, Ellen Scotton, Débora Soligo Fracasso, Adriane Ribeiro da Rosa, Catia Santos Branco, Mirian Salvador

**Affiliations:** ^1^Instituto de Biotecnologia, Universidade de Caxias do Sul, Caxias do Sul, RS 95070 560, Brazil; ^2^Laboratório de Psiquiatria Molecular, Hospital de Clínicas de Porto Alegre, Porto Alegre, RS, Brazil; ^3^Programa de Pós-Graduação em Farmacologia e Terapêutica, Universidade Federal do Rio Grande do Sul, Porto Alegre, RS, Brazil

## Abstract

The prevalence of psychiatric disorders has increased in recent years. Among existing mental disorders, major depressive disorder (MDD) has emerged as one of the leading causes of disability worldwide, affecting individuals throughout their lives. Currently, MDD affects 15% of adults in the Americas. Over the past 50 years, pharmacotherapy, psychotherapy, and brain stimulation have been used to treat MDD. The most common approach is still pharmacotherapy; however, studies show that about 40% of patients are refractory to existing treatments. Although the monoamine hypothesis has been widely accepted as a molecular mechanism to explain the etiology of depression, its relationship with other biochemical phenomena remains only partially understood. This is the case of the link between MDD and inflammation, mitochondrial dysfunction, and oxidative stress. Studies have found that depressive patients usually exhibit altered inflammatory markers, mitochondrial membrane depolarization, oxidized mitochondrial DNA, and thus high levels of both central and peripheral reactive oxygen species (ROS). The effect of antidepressants on these events remains unclear. Nevertheless, the effects of ROS on the brain are well known, including lipid peroxidation of neuronal membranes, accumulation of peroxidation products in neurons, protein and DNA damage, reduced antioxidant defenses, apoptosis induction, and neuroinflammation. Antioxidants such as ascorbic acid, tocopherols, and coenzyme Q have shown promise in some depressive patients, but without consensus on their efficacy. Hence, this paper provides a review of MDD and its association with inflammation, mitochondrial dysfunction, and oxidative stress and is aimed at thoroughly discussing the putative links between these events, which may contribute to the design and development of new therapeutic approaches for patients.

## 1. Introduction

Major depressive disorder (MDD) is a public health problem characterized as a mental disorder and is one of the leading causes of occupational or social disability worldwide. According to the World Health Organization [1], 322 million people are affected by this disorder, which is currently more predominant among women than men.

First-line treatment for depression includes talk therapies, antidepressant medications, or a combination of both. Patients suffering from mild depression are indicated for cognitive behavioral therapy, while for moderate to severe cases, antidepressants are indicated [1]. The full benefit of the medications occurs 4 to 6 weeks after initiation of administration [2].

Less than half of patients worldwide (in many countries, representing less than 10%) receive these treatments. In addition, other difficulties include lack of resources and/or skilled professionals, diversity of clinical manifestations, social stigma associated with mental disorders, and inaccurate assessment [1]. Despite the approaches available to treat MDD, only about one-third of depressed patients achieve remission upon receiving antidepressant treatment, and treatment response rates appear to drop with each subsequent retry [3, 4].

Currently available antidepressant therapies focus on modulating monoamine transmission, or they may limit it, as depression is a very broad disease and involves a sequence of events, and monoamine medications do not have a wide range of options. To assist the large number of refractory patients in recent years, the addition of atypical antipsychotics to antidepressants has been common and has some benefit [5]. Nevertheless, many patients continue to suffer from this disabling disease.

Treatment-resistant depression (TRD) is associated with increased functional impairment, mortality, morbidity, and long-term recurrent or chronic episodes [6, 7]. Therefore, an improved response to treatment by identifying predictive risk factors for nonresponse may help better disease prognosis [8].

Major depressive disorder has been associated with alterations in neurotransmitter biosynthesis, altered membrane receptor expression, alterations in cortical structure volume, and desensitization of the hypothalamic-adrenal-pituitary (HPA) axis [9]. HPA axis dysregulation causes excessive release of cortisol, a fundamental hormone for maintaining homeostasis, as it has numerous catabolic functions and anti-inflammatory action. However, its excessive production can suppress the immune system [10]; thus, inflammatory responses are triggered through the activation of macrophages and lymphocytes, as well as microglia and astrocytes [11]. The first studies on depression date back to the 1980s, and since then, the findings show that inflammation could play an important role in the pathophysiology of this disease [12–14].

In fact, several studies have shown changes in interleukin-6 (IL-6), tumor necrosis factor alpha (TNF-*α*), interferon gamma (IFN-*γ*), and C-reactive protein (CRP) levels [15–17] in depressed patients. Some of these cytokines activate the enzymes indoleamine 2,3-dioxygenase (IDO) and tryptophan 2,3-dioxygenase (TDO) from the kynurenine pathway, diverting tryptophan from its main route of serotonin production [18–20].

A growing body of evidence [21] indicates that inflammation may further cause deleterious changes in mitochondrial function, affecting oxidative phosphorylation and membrane polarity. These changes may lead to oxidative and nitrosative stress and apoptosis, events associated with the pathogenesis and pharmacological resistance of MDD [18, 21–23]. Thus, mitochondria should be considered a crucial target for the development of new antidepressant drugs, and specific forms of mitochondrial dysfunction can be identified as biomarkers to customize treatment and aid in early diagnosis [21]. In this paper, we discuss the involvement of inflammation, mitochondrial dysfunction, and oxidative stress in the etiology and pharmacological resistance of MDD as pathways for future therapeutic approaches.

## 2. Methodological Approach

**Figure 1:**
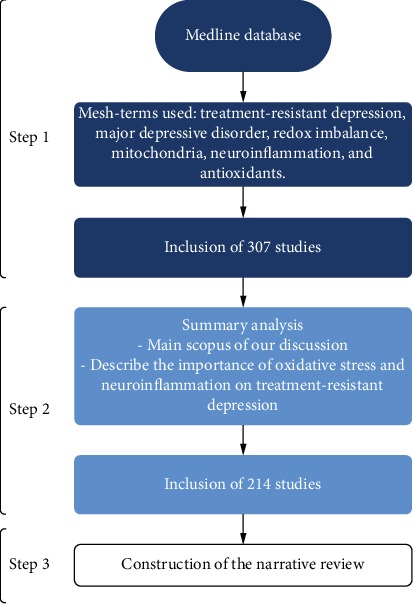
Flowchart of the search methodology performed.

Three hundred and seven (307) articles were selected from the search in the MEDLINE database. We used a combination of one or more of the following mesh-terms: treatment-resistant depression, major depressive disorder, redox imbalance, mitochondria, neuroinflammation, and antioxidants. The terms cited must appear in the title, keywords, or abstract of the article. After this search, we analyzed the abstract of each article and only those that contemplated the main scope of our discussion and could help us to describe the importance of oxidative stress and neuroinflammation on treatment-resistant depression were selected. Thus, 214 studies were included, which were analyzed and used to construct the rationale of our narrative review.  Figure 1 illustrates the search methodology.

## 3. Inflammatory Hypothesis of Depression: HPA Axis Desensitization

Acute activation of the HPA axis plays a crucial role in responding appropriately to acute stress events. However, prolonged activation of the axis and a sustained increase of glucocorticoids are well documented and related to MDD [24]. Depressive individuals have an increase in plasma cortisol concentration and a decrease in HPA axis sensitivity to dexamethasone [25, 26]. Briefly, the alpha glucocorticoid receptor (GR*α*) mediates the negative feedback of the HPA axis, i.e., the ability of the cortisol to inhibit its secretion. In contrast, in depression and chronic stress situations, a decrease in GR*α* expression in the hypothalamus and pituitary leads to the desensitization of negative feedback, which in turn leads to HPA axis hyperactivity and a sustained increase in synthesis and secretion of glucocorticoids [27, 28].

The lack of adequate glucocorticoid-mediated inhibitory control promotes increased immune signaling, as demonstrated by increased levels of cytokines and proinflammatory cells activated by glucocorticoids [16, 29]. Lymphocytes from patients with MDD are also resistant to the suppressive effects of dexamethasone in vitro [30]. Glucocorticoid resistance and an intensification of proinflammatory signaling are found in about 85% of MDD studies [31]. Proinflammatory cytokines are also involved in glucocorticoid resistance by decreasing GR expression and function, leading to a pronounced increase in inflammatory responses [32]. These studies confirm that glucocorticoid resistance increases cortisol production and increased inflammatory signaling, which are coexisting biological responses and influence the therapeutic response in depression.

The antidepressant treatment enhances the synthesis of brain-derived neurotrophic factor (BNDF) and promotes neurogenesis. However, drug actions appear to depend on glutathione reductase (GR) activation, and glucocorticoid activation of GR*α* decreases neurogenesis. This contradiction may be related to the different effects of GR*α* due to its binding to different agonists and modifications of their phosphorylation state. Different antidepressants act on the glucocorticoid receptor and increase neurogenesis through mechanisms dependent on the activation of protein kinase A (PKA) and its signaling cascade [33]. However, cortisol decreases neurogenesis through upregulation of the kinase gene induced by serum and glucocorticoids (SGK1) [34]. This in vitro evidence demonstrates PKA antidepressant-induced positive regulation and cortisol-induced expression of SGK1 [34, 35]. These data indicate that persistent signaling of glucocorticoids and norepinephrine, in a situation of psychosocial stress, trauma, or persistent stressful events, contributes to exaggerated inflammation responses, desensitizing GR*α* and activating transcription of proinflammatory genes.

Peripherally released cytokines can reach the central nervous system (CNS) through three distinct pathways: the blood-brain barrier, the circumventricular organs, or the vagus nucleus of the solitary tract. When cytokines reach the CNS, they directly and indirectly affect the metabolism of neurotransmitters, which can stimulate apoptosis and decrease neurogenesis, affecting essential circuits for behavior maintenance. They alter the metabolism of serotonin, dopamine, and glutamate, which are neurotransmitters involved in mood regulation [16]. As serotonin levels decline, production of melatonin is impaired, which, in turn, disrupts the biological clock that controls neuronal physiological processes, including the sleep-wake cycle [36, 37]. This combination of factors accentuates depressive symptoms, as well as oxidative stress and inflammation in the CNS [38, 39]. A promising drug for treating circadian rhythm in psychiatric diseases is ramelteon (RMT), a melatonin receptor agonist [40]. Previous evidence indicates that RMT has neuroprotective, antioxidant, and anti-inflammatory activities [41].

Controlling inflammation in MDD patients is crucial. In response to stress, the innate immune system activates the “sterile inflammatory response” which releases molecules into the extracellular space. The Danger-Associated Molecular Pattern (DAMP) binds to Pattern Recognition Receptors (PRRs) expressed on the cytosol or innate immune cell membranes that may be NOD (NLR) or Toll (TLR) receptors. The cascade triggering of these PRRs leads to the activation of NLRP3 inflammasome and caspase-1 [42]. NLRP3 in turn activates IL-6, TNF-*α*, and IFN-*γ*, which may increase the activity of indoleamine 2,3-dioxygenase (IDO), an enzyme involved in the synthesis of kynurenine (KYN) from the amino acid tryptophan (TRP) [43, 44].

**Figure 2:**
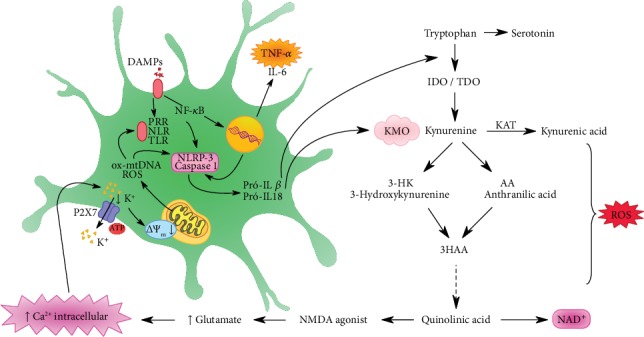
Danger-Associated Molecular Pattern (DAMP) binds to the Pattern Recognition Receptors (PRRs) expressed on the cytosol or in innate immune cell membranes. The cascade triggered by these PRRs leads to NLRP3 inflammasome and caspase-1 activation, which can activate IL-1*β* and IL-18. Oxidized mitochondrial DNA (ox-mtDNA) and mitochondrial reactive oxygen species (ROS) also activate the inflammasome. NF-*κ*B, through the transcriptional activation pathway, generates tumor necrosis factor alpha (TNF-*α*) and interleukin-6 (IL-6). Proinflammatory cytokines IL-1*β* and IL-18 activate the enzymes IDO and TDO of the kynurenine pathway, degrading tryptophan into kynurenine. These two cytokines further activate KMO, which is the enzyme that directs kynurenine to be degraded to 3HK and quinolinic acid, both neurotoxic agents, over the kynurenic acid, a neuroprotective agent. Kynurenic acid is an NMDA receptor agonist and increases glutamate levels and consequently intracellular calcium. Excessive amounts of ROS are produced over the kynurenine pathway.

Tryptophan is an essential amino acid and is the primary precursor of serotonin. Activation of IDO reduces serotonin synthesis through a shift of tryptophan to the kynurenine pathway. In the brain, kynurenine is metabolized by the following cellular pathways: (1) neural progenitor cells and microglia, generating 3-hydroxykynurenine (3-HK) and quinolinic acid (QA), and (2) astrocytes, producing kynurenic acid (KA). The metabolite, 3-HK, is involved in oxidative stress. QA is an N-methyl-d-aspartate (NMDA) receptor agonist that promotes the increased release of glutamate and blocking its reuptake by astrocytes [45]. Increased glutamate is known to cause neurotoxicity because excess neurotransmitter triggers an influx of Ca^2+^ into the cell, which in turn can generate a potassium leak in the cell. This depolarizes the mitochondrial membrane, generating ROS and oxidized mitochondrial DNA (ox-mtDNA), which reactivates NLRP3 [46, 47]. Influx of Ca^2+^ may also activate other pathways that lead to mitochondrial dysfunction by reducing SIRT3 (Figure 2). This set of results can lead to excitotoxicity and neurodegeneration in essential brain areas of depressed individuals (Figure 2). In fact, an increase in QA concentration in the brain and cerebrospinal fluid of depressive individuals suffering from suicide was already found [48, 49]. In addition, increased QA concentration has been associated with oxidative stress and lipid peroxidation [50].

The reduction in serotonin synthesis by cytokines may also reduce dopamine synthesis. Injection of IFN-*γ* into rats resulted in a decreased concentration of tetrahydrobiopterin (BH4) and dopamine in the amygdala and raphe nuclei [51]. The BH4 is an essential cofactor for the regular activity of the enzyme tyrosine hydroxylase, which is the critical enzyme in the dopamine biosynthesis pathway [16]. The impact of cytokines on reducing synthesis and dopaminergic action in the brain can lead to a decreased motivation and pleasure (anhedonia), an essential and classic symptom of depressive behavior.

### 3.1. Neuroinflammation and the Role of Microglia

Some clinical studies suggest that microglia have a modified morphology and function in depressive individuals, with a less branched phenotype and less capacity for glutamate reuptake and maintenance of homeostasis [52, 53]. Moreover, in a study using positron emission tomography, individuals who presented a depressive episode demonstrated an increase in translocating protein (TSPO) labeling, which is a neuronal marker of inflammation [54]. Postmortem studies found an increase in the expression of cytokines and complement pathways in the prefrontal cortex and hippocampus of depressive individuals [55, 56], suggesting that neuroimmune dysregulation may represent a pathophysiological mechanism in depressive patients.

Changes in neuronal functions that occur concurrently with microglial activation imply reciprocal interactions between these two structures, and these responses may not only lead to neuroinflammation but also affect other essential functions of the CNS, such as neurotransmission. Morphological changes in microglia induced by neuroinflammation generally do not lead to acute neurotoxicity but may contribute to neuronal dystrophy after stress. Furthermore, exposure to psychosocial and environmental stress causes neuronal activation and release of glutamate and norepinephrine in corticolimbic brain regions, such as the prefrontal cortex, amygdala, and hippocampus [57]. This evidence suggests that dysfunctional neuronal activation associated with increased glucocorticoids leads to neuronal dystrophy in corticolimbic brain regions following chronic exposure to stress. For example, repeated stress caused dendritic atrophy and loss of synapses on pyramidal neurons in the prefrontal cortex of rats [58, 59], and these effects were also achieved after chronic corticosterone administration [60]. These neurobiological alterations contribute to the change in excitatory-inhibitory activity in the prefrontal cortex, a critical region for the maintenance and control of motivated behaviors, which is critically affected in depression [61, 62].

The increased release of DAMPs from the high mobility moiety (HMGB1) box 1 protein caused the activation of microglia and increased gene expression of proinflammatory cytokines [63]. Studies suggest that increased NLRP3 and IL-1*β* activation in the prefrontal cortex are mediated by the activation of microglia in chronic stress situations and that these responses are reversed under chronic treatment with fluoxetine [64].

Recent studies have also demonstrated that anxiolytics and antidepressants can block or reverse microglial activation. Administration of imipramine during a social defeat protocol reduced IL-6 expression in microglia and attenuated depressive-like behavior [65]. This result is similar to other studies, which found that selective serotonin reuptake inhibitors produce an anti-inflammatory response in microglia [66]. These findings raise important questions. (1) Do the interventions mentioned above aimed at normalizing monoamine neurotransmission exert their primary effects through neurotransmitter homeostasis? (2) Or can the inhibition of microglial activation be considered their primary mechanism of action? These results suggest that the pharmacological treatments currently used against depression may produce their therapeutic effects by enhancing neurotransmission and modulating microglial functions and inflammation. Understanding neuronal function and microglia and the distinction of molecular and cellular pathways that contribute to the maintenance of neuronal and microglial function after repeated exposure to stress may provide new insights into potential therapeutic targets.

Additional studies must be conducted to examine the relationships between microglia and neurons and, at the same time, to find out if the interaction between these cells contribute to the stress-induced inflammation. In addition, how can interventions involve these mechanisms to provide effective therapeutic benefits?

### 3.2. Inflammation and Treatment Resistance

The development of biomarkers capable of predicting the response to depression treatment is clinically important. These biomarkers could be critical for classifying patient subtypes and a good alternative for prescribing specific treatments if patients are resistant to the conventional antidepressant drug.

A recent review by Roman and Irwin [67] describes that proinflammatory cytokines (mainly IL-1*β*, IL-6, and TNF-*α*) may be useful biomarkers for investigating the presence or level of baseline inflammation during screening for depression. However, the choice of these immune biomarkers is limited due to the lack of standardization of assays for clinical application. The authors also cite a study in progress by Janssen Research & Development, LLC (ClinicalTrials.gov Identifier: NCT02902601), which is investigating the safety and tolerability of a new agent, JNJ 54175446, in patients with depression. JNJ 54175446 is a P2X7 purinergic receptor agonist capable of indirectly modulating inflammasome activation.

Despite decades of research about depression, we still lack a deeper understanding of the pathophysiology and mechanisms involved in drug resistance. In other areas, such as in diabetes or heart disease [68], a large genomic association between these illnesses and the molecular targets of marketed drugs is known, with high treatment efficacy. Unfortunately, the situation is entirely different in psychiatry, where none of the currently used drugs has a strong correlation with potential candidate biomarkers for Genome-wide Association Studies (GWAS) [69]. The genetic risk variants identified so far cover a broad spectrum of biological processes but are enriched for neurodevelopmental or synapse-related genes and are not directly related to the targets of current clinical medications. Taken together, these findings point to new avenues for antidepressant therapy, suggesting entirely new biology for these disorders and the urgent need to reconsider other factors involved with depression and drug resistance.

Many clinical factors have been related to the nontherapeutic response, including psychiatric comorbidities such as anxiety disorders, personality disorders, and bipolar disorder [70–72]. In addition, comorbidities of cardiovascular diseases, diabetes, and cancer have also been associated with an inefficient response to antidepressants [73]. Interestingly, these clinical conditions are associated with inflammation [16, 45, 74]. Investigation of the pathways involved with the pathophysiology of MDD has produced numerous promising therapeutic targets for treatment-resistant depression (TRD). Among these new markers, there is a particular interest in the inflammatory pathways and their link with oxidative and nitrosative stress [75–79].

We will now explore data about how inflammation and the outcomes that are related to it may influence the response to antidepressant treatment. A meta-analysis of 35 studies evaluating inflammatory markers before and after antidepressant treatment found that patients who were not responders were more likely to have a higher inflammatory profile at baseline and follow-up treatment than the responders [8]. In a study by Lindqvist et al., IL-6 decreased significantly in patients responding to selective serotonin reuptake inhibitors but did not reduce their concentrations in patients refractory to the treatment [80]. In a study of 241 depressive patients, Uhrer et al. indicated that the concentration of C-reactive protein (CRP) promoted a differentially predicted response to escitalopram and nortriptyline. Other studies found higher levels of CRP and other proinflammatory cytokines early in the study, and these inflammatory markers were associated with a weaker response to serotonin reuptake inhibitor antidepressants, including escitalopram, fluoxetine, and a low dosage of venlafaxine [81]. In a study by Haroon et al., patients who participated in multiple treatment trials and who failed symptom remission exhibited higher plasma concentrations of TNF-*γ*, sTNFR2, IL-6, and CRP compared to depressive individuals who responded efficiently. Following a body mass index (BMI) correction criterion, TNF-*α*, sTNF-R2, and IL-6 were the markers most associated with the number of failed treatment attempts [82].

Preclinical and clinical studies suggest that a decrease in NMDA receptor activity and an increase in AMPA receptors lead to favorable mood outcomes [83]. Increased stimulation of AMPA over NMDA receptors leads to an increase in calcium and sodium influx and a strengthening of intracellular signaling that enhances BDNF expression, leading to improved neuroplasticity and neuronal function [73, 77, 78]. Thus, increased AMPA activity and decreased NMDA activity may be essential outcomes for mood enhancement and cognition and promising targets for TRD [79, 83–85].

Using a preclinical model of inflammation, administration of lipopolysaccharide (LPS) has been shown to induce depressive-like behavior through NMDA receptor stimulation [86]. Briefly, LPS induces a depressive-like behavior by activating IDO [87], leading to an increase in the KYN/TRP ratio and an increase in the formation of QA [88], an agonist of the NMDA receptor. The discovery reinforced the importance of the NMDA receptor for depression influenced by inflammation in which ketamine improves the signs of distress by strengthening glutamatergic neurotransmission through AMPA receptors [86]. The use of ketamine for other purposes, including the treatment of MDD, has been studied [89]. Several studies have already shown an improvement in antidepressant response, including for patients with TRD. These findings will be discussed below in the section on new therapeutic perspectives.

The information above suggests that a portion of depressive patients has a high inflammatory profile and that such inflammation may be associated with failure in multiple treatment attempts. Thus, in patients with a history of failed antidepressant treatment, clinicians could evaluate and consider the use of therapies that act on the inflammatory profile or molecules that are related to inflammation.

## 4. Adjuvant Therapies

Many studies using TNF-*α* antagonists (infliximab); nonsteroidal anti-inflammatory drugs (NSAIDs); ketamine, natural anti-inflammatory agents, such as omega-3 polyunsaturated fatty acid and curcumin; and an NMDA receptor antagonist have gained attention. Among the aforementioned agents, infliximab has been the most evaluated for the treatment of TRD.

### 4.1. TNF-*α* Antagonist (Infliximab)

The TNF-*α* antagonists are used clinically in autoimmune disorders to prevent a systemic inflammatory response. The reduction in microglia activation with the use of TNF-*α* antagonists may be particularly relevant for TRD [90]. A recent meta-analysis (*n* = 2370) of seven randomized controlled trials using anticytokine agents (e.g., adalimumab, etanercept, tocilizumab, and infliximab) in conditions of chronic inflammation (e.g., rheumatoid arthritis) reported moderate antidepressant efficacy (standardized mean difference (SMD) = 0.40, 95% confidence interval (CI) = 0.22, 0.59) [91].

The antidepressive efficacy of infliximab alone has been investigated and reported as a primary outcome among adults with mood disorders. This randomized controlled clinical trial tested the efficacy of intravenous infliximab (5 mg/kg) administered at baseline and weeks 2, 4, and 6 of a 12-week protocol in a sample of 60 patients with TRD. The study found no difference between the treated and control groups; however, when patients were categorized for their inflammatory status based on (PCR) > 5 mg/L, a significant antidepressant effect was noted. Sixty-two percent of patients with TRD who received infliximab achieved a 50% reduction in HDRS scores, compared to only 33% of patients in the placebo group [92].

Although infliximab may be useful for a subset of patients with TRD, who also have increased inflammatory biomarkers, further studies are needed to confirm or refute this hypothesis. In addition, the number of adverse effects may be a limiting factor, as infliximab increases the risk of infection due to its potent anti-inflammatory effect.

### 4.2. Nonsteroidal Anti-Inflammatory Drugs (NSAIDs)

Proinflammatory cytokines can trigger an inflammatory cascade in the brain, which includes increased activity of cyclooxygenase (COXs) that are critical enzymes in the production of prostaglandins [93]. Drugs targeting the cyclooxygenase-1 (COX-1) and cyclooxygenase-2 (COX-2) enzymes might have a beneficial effect on depressive patients with elevated levels of inflammatory cytokines. COX-2 expression is detected in synaptic dendrites and excitatory postsynaptic endings, especially on the cortex, hippocampus, and amygdala, whereas COX-1 is expressed in microglia and perivascular cells [94]. A study by Choi et al. [95] demonstrated that mice with COX-1 deficiency had a decrease in neuronal degeneration, microglial activation, and expression of proinflammatory cytokines and PGE2 after exposure to LPS.

However, COX-2 may play a neurotoxic or anti-inflammatory role, depending on the initial stimulus. The results of studies using animal models, especially with celecoxib treatment (a selective COX-2 inhibitor), are contradictory. In a chronic mild stress model in rats, treatment with celecoxib for 21 days reversed depressive behavior [96]. In another preclinical model of depressive-like behavior (bulbectomy), the use of celecoxib for 14 days reversed depressive behavior in treated rats. The concentration of the proinflammatory cytokines IL-1 and TNF-*α* in the prefrontal cortex and hypothalamus decreased, probably due to the reduction of PGE2 synthesis [97]. However, COX-2 may also have a neuroprotective function in response to an inflammatory challenge. Deletion of COX-2 may result in increased neuronal damage in the hippocampus and increased expression of TNF-*α*, IL-6, and IL-1*β*. Chronic administration of celecoxib for six weeks caused an increase in IL-1*β* levels in the brain of mice exposed to LPS [98].

NSAIDs, especially acetylsalicylic acid (ASA) and celecoxib, were also tested in an attempt to improve response in patients with TRD. Acetylsalicylic acid irreversibly inhibits COX-1 and COX-2, thus decreasing prostaglandin and thromboxane levels, and the production of TNF-*α* and IL-6 [99]. In 2013, Berk et al. published a systematic review that evaluated the role of ASA in the treatment of mental illness, evaluating studies from 1996 to 2012 [100]. Some evidence suggests beneficial effects for aspirin in mood disorders, through an improvement in the clinical response time to antidepressants [101]. That study found that patients who used fluoxetine associated with ASA had a higher rate of remission for depressive symptoms than the fluoxetine group. In another study with 70 depressive patients, administration of aspirin in combination with fluoxetine conferred a further reduction of oxidative stress parameters compared to fluoxetine monotherapy [102]. Despite the lack of significant clinical improvement and linear scientific data, NSAIDs act positively on many of the biochemical and molecular outcomes cited in the text. However, the use of NSAIDs chronically provides significant adverse effects to be considered and does not demonstrate a congruency in the results.

### 4.3. Ketamine: NMDA Antagonist

Interest in using ketamine to treat TRD has increased in the last decade. The long-term beneficial actions of ketamine on the central nervous system are very evident; however, there are not enough studies to discuss the long-term effects of a single ketamine administration on the functioning of neural circuits in MDD [103]. Previous evidence demonstrates that ketamine may increase the expression and synthesis of BDNF [103, 104]. The antidepressant effects of ketamine were inhibited in mice with BDNF deletion as well as after infusion of an anti-BDNF antibody into the prefrontal cortex [105]. Furthermore, preclinical studies show that ketamine exerts its antidepressant effects by increasing the expression of mammalian rapamycin target protein (mTOR), which modulates cell growth, proliferation, motility, survival, and protein synthesis [103, 106]. These observations provide insight into why the decreased levels of BDNF associated with depressive symptoms could be reversed through the rapid actions of ketamine. Several clinical studies have tested ketamine as an antidepressant agent for TRD.

In a recent meta-analysis [107], seven randomized controlled trials (RCTs) using intravenous infusion and intranasal ketamine evaluated its antidepressant effect in patients with MDD and bipolar depression. Ketamine was associated with higher clinical remission rates in the control groups (saline or midazolam) 24 hours (OR 7.06, NNT = 5), three days (OR 3.86, NNT = 6), and seven days (OR 4.00, NNT = 6) after remission. Ketamine is associated with transient psychotomimetic effects; however, these signs do not persist chronically [107].

In addition, several clinical trials were conducted to evaluate the role of ketamine in TRD. In a randomized, placebo-controlled, double-blind, crossover study, 18 patients with TRD received an infusion of ketamine (0.5 mg/kg) with a one-week interval between each administration. Patients who received ketamine obtain significant improvement in evaluations compared to placebo subjects. The positive effect was apparent within 110 minutes after injection and remained over the following days [108]. In other clinical studies, similar results have been observed [109, 110]. In two case-control studies, ketamine had an antisuicidal effect in the TRD subjects [111, 112].

Despite the promising effects of ketamine in patients with TRD, opinions about its use to treat MDD are still polarized, mainly because of its side effects. Some of the most common effects are dissociations related to depersonalization, as well as perceptual disturbances and mental confusion. The risk for the development of psychotic states, agitation, and dependence is also referred to as an adverse effect [113]. Nevertheless, ketamine is clearly a potent, rapid-acting antidepressant for TRD, which is entirely different from conventional antidepressants that take days to weeks before a crucial antidepressant effect is observed [114]. Therefore, ketamine may be a useful alternative for TRD. ESKETAMINE (ESK), its derivative, is a new NMDA drug recently approved by the FDA for intranasal use in TRD treatment. A recent systematic review reported that the intravenous infusion of ESK causes rapid and sustained antidepressant activity in refractory patients with MDD and TRD, as well as in patients with MDD at imminent risk of suicide [115]. However, despite its effectiveness, further preclinical and clinical studies are needed to investigate the long-term efficacy and safety of intranasal ESK. Another recent FDA-approved drug for treating TRD is Symbyax, which combines olanzapine (an atypical antipsychotic) and fluoxetine (a selective serotonin reuptake inhibitor) [116, 117]. A recent study of 25 patients treated with Symbyax for 8 weeks showed a decline in amygdala activity and right ventromedial prefrontal metabolism, and these events were correlated with improvement in depression after the intervention [118].

## 5. The Relation between Inflammation and Oxidative Stress

In the brain, oxidative stress and its related cellular damage are easily spread due to the physiological and physical characteristics of this organ, as well as the high metabolic rate of its cells, which make it highly dependent on an efficient mitochondrial oxidative phosphorylation system (OXPHOS). Mitochondria are intracellular organelles required for numerous cellular functions, including control of energy metabolism and regulation of ROS production, calcium homeostasis, and apoptosis. During the inflammatory process, released interleukins are capable of activating the KYN pathway, which generates catabolites, called TRYCATs that cause a high calcium influx inducing mitochondrial dysfunction along with an impairment in the cellular antioxidant system [119, 120]. This becomes a cycle as the mitochondrial dysfunction and potential losses of the mitochondrial membrane lead to a rapid increase in the production of mitochondrial reactive oxygen species (MROS), which are also activators of the inflammasome (NLRP3) [121] (Figure 2).

The macrophages involved during the inflammatory process have a P2X7 receptor (P2X7R), which is a cationic channel highly dependent on exogenous ATP. Activation of this channel causes potassium efflux, altering mitochondrial membrane potential and generating mtROS and oxidized mitochondrial DNA, which are released into the cytosol by directly activating NLRP3 [46]. Potassium efflux may also occur due to high levels of calcium from the kynurenine pathway (Figure 2).

The increase in ROS production is associated with a reduction in neuronal metabolism. This metabolic worsening may be associated with the reduced ability to convert external energy into substrates required for ATP biosynthesis, mainly due to mitochondrial dysfunction [122]. Metabolic stress and increased ROS production can lead to advanced glycation end products (AGEs) and lipid peroxidation end products in neurons [123].

A study by Anamika et al. reports that influx of Ca^2+^ activates neural nitric oxide synthase (nNOS) and nitric oxide release occurs. This nitric oxide can be combined with other types of ROS to form peroxynitrite (ONOO-), a highly unstable radical [124]. Excess nitric oxide may decrease SIRT3 activity and expression, causing mitochondrial dysfunction [125].

Sirtuins are NAD+-dependent histone deacetylases. Because they require NAD+ for their activity, the cellular level of sirtuins represents the redox status of cells and, thus, serves as metabolic stress sensors [125]. SIRT3 in particular is an isoform present in mitochondria, whose function is to modulate activity of several important mitochondrial proteins, to induce adaptive changes during bioenergetic deficits, and to regulate mitochondrial biogenesis and dynamics, ROS metabolism, ATP production, and maintenance of mitochondrial integrity [126, 127].

SIRT3 is also related to mitochondrial permeability transition pore (mPTP) blockade of the pores, preventing the release of cytochrome C and thus preventing apoptosis [125, 128]. All these changes can occur from the reduction of SIRT3, including low ATP production, which is very common in neurodegenerative diseases, including depressive patients [125].

With the reduction of SIRT3, the antioxidant defense mechanism also changes, since SIRT3 deacetylates the antioxidant enzyme MnSOD, and mitochondria are the main site of ROS generation, reducing antioxidant the immediate target of oxidation damage [129, 130]. The enzyme MnSOD catalyzes the conversion of superoxide anions (O^−2^) into hydrogen peroxide (H_2_O_2_) and oxygen (O_2_). Reduced MnSOD activity is the immediate target of oxidation damage [129, 130].

SIRT3 regulates the tricarboxylic acid cycle (TCA) by activating PDH (pyruvate dehydrogenase complex), the first enzyme that catalyzes the entry of pyruvate into the pathway of oxidative energy production [131]. In addition, SIRT3 can deacetylate mitochondrial complex I, specifically subcomplex 9 (NDUFA9) [132, 133]. SIRT3 is likely to regulate the expression of some complex IV subunits and is also related to the two subunits of ATP synthase F0-F1 [126, 134, 135]. Thus, inflammation generated by HPA axis imbalance leads to mitochondrial dysfunction, which in turn causes excessive ROS production.

**Figure 3:**
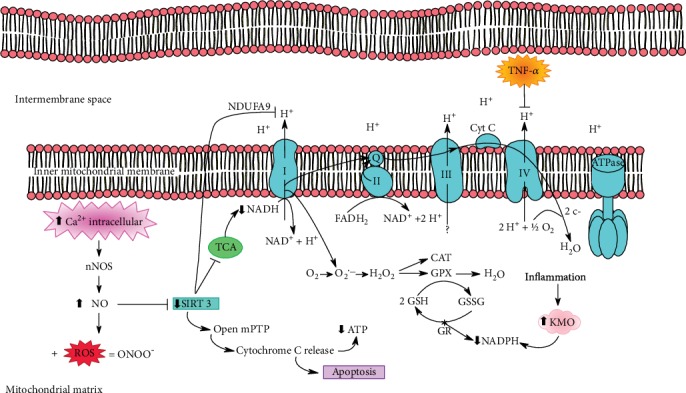
The increase of intracellular calcium activates neural nitric oxide synthase (nNOS), causing increased NO levels. This will decrease SIRT3, which acts as a key to control mitochondrial dysfunction. As SIRT3 activity decreases, mitochondrial permeability transition pores (mPTPs) open, which release cytochrome C, causing a decrease in ATP levels and inducing apoptosis. NO can also bind to ROS from the kynurenine pathway, generating peroxynitrite (ONOO), a highly unstable free radical. Reduction of SIRT3 deacetylates complex I NADH dehydrogenase, specifically in the NDUFA9 subunit, which interacts with two other ATP synthase subunits (F0 and F1). When SIRT3 is reduced, PDH activation is inadequate for the citric acid cycle, resulting in low levels of NADH and reduced activity of complex I. In addition, SIRT3 promotes deacetylation of MnSOD, an antioxidant enzyme that scavenges superoxide anion produced over the pathway. Another impaired antioxidant enzyme is GSH, because during the inflammatory process, the enzyme KMO, an enzyme dependent on NADPH, is activated, thus reducing the availability of this coenzyme for antioxidant defense systems. In parallel, TNF-*α* phosphorylates tyrosine 304 in subunit I of cytochrome C oxidase in complex IV, leading to further mitochondrial damage.

A redox imbalance in the brain might be involved in the pathogenesis of depression beyond being related to other risk factors such as increased inflammation, impaired plasticity, and reducing neuron signaling [136]. Under physiological conditions, ROS and reactive nitrogen species (RNS) are regulated by antioxidant pathways, including enzymatic and nonenzymatic compounds. In excess or in situations where the antioxidant system is impaired, these species may damage lipids, proteins, and DNA. In depressed patients, the antioxidant system is also compromised because inflammation activates the KMO (kynurenine 3-monooxygenase) enzyme that degrades kynurenine into 3-hydroxykynurenine and other neurotoxic metabolites. The KMO enzyme is NADPH-dependent, so if the availability of this coenzyme is reduced, NADPH-dependent antioxidant defense systems are compromised, including glutathione (GSH) and catalase (CAT), which are key enzymes for reducing H_2_O_2_ in O_2_ and H_2_O [137] (Figure 3).

MDD is usually accompanied by a decrease in antioxidant enzyme activities and total antioxidant capacity (TAC). A recent meta-analysis identified lower TAC and some antioxidant parameters in acute episodes of depressed patients, as well as serum paraoxonase, uric acid, albumin, and zinc levels. On the other hand, higher oxidative damage products, including red blood cell (RBC) malondialdehyde, serum MDA and 8-F2-isoprostanes, and serum peroxide were found in MDD patients [138].  Table 1 summarizes oxidative/antioxidative markers in drug-naive patients with depression.

In the oxidative stress pathway, superoxide dismutase (SOD) is the primary antioxidant enzyme able to protect cells from damage caused by ROS. Disturbances in SOD activity are usually found in depressive patients, but the findings are still inconsistent with respect to the direction of this disruption [154]. Decreased SOD activity has been identified in MDD patients [148]. However, other studies reported increased SOD and catalase (CAT) activity in MDD patients [151]. Recently, Tsai and Huang found that serum SOD and CAT activities were significantly higher in the acute phase of MDD patients, suggesting that increased activities of both antioxidant enzymes might be indicators of acute depressive episodes on MDD [143].

Glutathione (GSH) is the most abundant low-molecular-weight thiol in the human body. It plays an important role in protecting cells and their components against ROS and represents a sensitive and consistently endogenous marker of oxidative stress [155]. The pathway that maintains intracellular GSH homeostasis comprises GSH redox cycling, which includes GSH oxidation by glutathione peroxidase (GPx) during detoxification of hydrogen peroxide (H_2_O_2_) or organic hydroperoxide and GSH reduction by glutathione reductase (GR) [155]. Peripheral measures in serum and plasma have already demonstrated a decrease in GPx activity in MDD patients [141]. In addition, negative correlations between GPx activity and severity of depressive symptoms were found, suggesting an impaired antioxidant protection in MDD [156]. Recently, a study using proton magnetic resonance spectroscopy to measure in vivo brain GSH in adolescents with MDD found low occipital GSH levels [141].

Production of H_2_O_2_ is balanced by catalytic action of antioxidant enzymes, such as CAT and GPx, and proteins with antioxidant function (peroxiredoxins) that act on H_2_O_2_ quickly and in synergy modulating the overall peroxide signal [157]. The transcription factor Nrf-2 (nuclear factor erythroid 2-related factor 2) is another target that seems to play an important role in redox homeostasis. At low oxidative stress levels, Nrf-2 is activated and stimulates the transcription of antioxidative genes, leading to the cytoprotective effects. Supporting the hypothesis that increased ROS plays a significant role in depression, Mellon et al. reported that genes regulated by Nrf-2 were elevated in MDD patients and decreased after effective antidepressant treatment [158]. In addition, a recent preclinical study with rodents found that vulnerability to depression resulted from a persistent state of oxidative stress, mediated by Nrf-2 dysfunction, which was reversed by treatment with antioxidants [159].

Several studies that evaluated lipid peroxidation in MDD patients suggest that increased serum levels of MDA, a product resulting from the reaction between ROS/RNS and lipids, are strongly associated with MDD [160]. A recent meta-analysis indicated that 8-hydroxy-2′-deoxyguanosine (8-OHdG) and F2-isoprostanes, which are measurements of oxidative DNA and lipid damage, respectively, are the two oxidative stress markers most consistently elevated in depression, usually with small to moderate effect sizes [144]. Increased MDA levels have also been found in patients with recurrent depression [148, 161, 162].

Increased levels of oxidative stress are also associated with larger cognitive impairment and one potential mechanism underlying neuroprogression and accelerated aging in mood disorders [163, 164]. Depression is known to be associated with accelerated brain aging [165]. One recent preclinical study that proposed a new model for aging brain found that oxidative stress at physiological levels may cause hippocampal dysfunction, specifically involving astrocytes, even before apoptosis detection [166]. Furthermore, a large body of evidence points to overproduction of ROS as one of the main causes of neuronal changes by inducing cell death and consequent atrophy of brain specific regions [167]. Smaller hippocampal volume has been associated with slower antidepressant response in late-life depression and increased peripheral oxidative stress in depressive patients [80, 168, 169]. All together, these findings may represent part of the interplay between oxidative stress, hippocampal volume, and treatment response.

The mechanisms of antidepressants against damage induced by excessive oxidative stress seem to be involved with remission of depressive symptoms and patients' recovery [136]. Evidence indicates that antidepressants may act to restore and normalize the activity of enzymes (such as SOD, GPx, GST, and GR) iNOS and xanthine oxidase (XO), increasing TAC levels, decreasing 8-OHdG and nitric oxide (NO) levels as well as lipid and protein oxidation and attenuating cell death induced by H_2_O_2_.  Table 2 shows the effects of antidepressant drugs on redox metabolism in clinical, preclinical, and mammalian cell culture models.

In general, antidepressants decrease oxidative damage markers in responsive patients, without altering this damage in nonresponsive patients (Table 2). However, there is no consensus about enzyme antioxidant defenses, as expected. Some studies found increased activity of antioxidant enzymes, while others observed a decrease. Most of these studies measured activity and nonexpression of enzymes, and this activity varies constantly and is not a static measure. Changes in enzyme activity are required to maintain or attempt to maintain cellular redox balance and occur in a matter of milliseconds. Studies with medicated depressive patients used different models, doses, and treatment times, which makes it difficult to compare their results. In addition, it remains unknown whether the effect of antidepressants on redox metabolism is direct or indirect or even a result of only improving the depressive state. Indirect effects can be mediated by different proteins, transcription factors, or anti-inflammatory effects. Thus, identifying the mechanisms responsible for the effects of antidepressants on redox metabolism is extremely important, because this is closely associated with the presence of symptoms in depressive patients.

Although many studies indicate increased lipid peroxidation in MDD, the first study was recently published that evaluated this parameter in TRD patients. Sowa-Kucma et al. found that TRD was accompanied by high levels of lipid peroxidation compared with non-TRD patients [189]. Another previous study investigated baseline OS markers (e.g., vitamin C and GPx) as predictors of antidepressant treatment response, finding negative results [190]. Other oxidative stress markers have been associated with poorer treatment response in MDD. Lindqvist et al. found higher baseline levels of F2-isoprostanes in nonresponders and a significant increase in 8-OHdG over the course of treatment in these patients [80].

Although antidepressant drugs have been shown to exert antioxidant effects in some study models (Table 2), it is unknown whether this is a direct or indirect effect of antidepressants. Together, lipid, protein, and DNA oxidative damage has been indicated as promising biomarkers for MDD patients under treatment; however, no clinical evidence for their use has yet been found. Nevertheless, there is evidence for the use of some antioxidants as a therapeutic approach for MDD. One promising candidate is N-acetylcysteine (NAC), a glutathione precursor that decreases inflammation and apoptosis, modulates levels of glutamate, promotes neurogenesis [191], and improves mitochondrial function [192]. These effects seem to be responsible for the remission of neurological symptoms in psychiatric diseases [193]. Berk et al. in a randomized carried 12-week study with a very expressive sampling (*n* = 269) reported antidepressant effects of NAC in patients with more severe depression (MADRS score of 25 or more) [194]. Furthermore, NAC exerts antioxidant effects in the anterior cingulate cortex of MDD patients in a multicenter RCT [195]. A recent study also described that chronic treatment with NAC improves depressive behavior and anxiety and spatial learning deficits as well as reverses pathological changes in the hippocampus in an animal model of neonatal depression [196].

Besides NAC, other antioxidants have been studied for their possible antidepressant effects. Recently, Abuelezz et al. reported that treatment with CoQ10 reversed depressive-like behavior, reduced lipid peroxidation, and restored GSSH and CAT levels in animals exposed to a chronic unpredictable mild stress (CUMS) protocol [197]. In addition, CoQ10 was able to recover the balance in kynurenine/serotonin levels by downregulating hippocampal IDO-1. Jahangard et al. found in a randomized study involving 89 patients with bipolar disorder, currently in a depressive episode, a significant increase in total thiol groups (TTG) and TAC, as well as a significant reduction in TNF-*α*, IL-10, and NO levels, after CoQ10 adjuvant therapy (200 mg/day) [198]. Corroborating with these findings, a decrease in CoQ10 levels was observed in TRD patients [153].

In turn, zinc (Zn) is an essential compound involved in several cellular processes and plays an important role against OS in the brain. Its neuroprotective properties include the blocking of excitotoxic Ca^2+^ influx and the upregulation of cellular antioxidant systems [199]. Clinical evidence points to an association of Zn deficiency and depressive behavior. According to the previous reports, Zn supplementation can improve mood in TRD patients [200]. Likewise, Zn administration increased BDNF expression and ameliorated the efficacy of antidepressant treatment, which can be relevant in the management of TRD [201, 202].

Curcumin, a potent anti-inflammatory plant metabolite, has been reported as a promising agent for the treatment of MDD [203–205]. Animal study models have already highlighted the potential of curcumin as an antidepressant [206, 207] with similar effects as fluoxetine and imipramine [208]. A meta-analysis that compared the use of curcumin with placebo demonstrated significant clinical efficacy of curcumin in improving depressive symptoms. Significant antianxiety effects were also reported [209]. Naqvi et al. demonstrated in a recent study using the CUMS protocol in rats that supplementation with curcumin (200 mg/kg) significantly attenuated the symptoms of depression and anxiety, reduced OS, and improved antioxidant status [210]. Curcumin also improved memory function and exhibited an inhibitory effect on acetylcholinesterase (AchE) activity. Similar results, demonstrated by Wang et al., showed that curcumin attenuated behavioral disorders associated with poststroke depression (PSD) in an animal model. These effects seem to be mediated by the control of Ca^2+^ levels in the CNS exhibited by curcumin [211].

Resveratrol (RES), another phenolic compound with recognized antioxidant activity, also has been studied as an antidepressant agent, but few studies have been conducted. Its beneficial effects on learning, memory, and anxiety [212] as well as on depressive-like behavior were reported [213]. Recently, in a study employing CUMS protocol, rats received RES (40 or 80 mg/kg/day) or fluoxetine (10 mg/kg/day) for four weeks. RES was able to reduce inflammation and apoptosis in the hippocampus and prefrontal cortex as well as expression of Akt/Akt and p-GSK3*β*/GSK3*β* proteins, achieving results similar to fluoxetine [214].

Considering all the evidence mentioned above, new investigations focusing on antidepressant effects of antioxidants or drugs with antioxidant activity should be performed, since the use of these compounds may emerge as a novel range of adjuvant therapy for resistant MDD management.

## 6. Limitations of the Current Research and Challenges Ahead

While this narrative review further supports that inflammation, mitochondrial dysfunction, and oxidative stress can be recognized as the central event in MDD, the question of why these events are still uncontrolled with conventional drug treatment remains unanswered. The few studies available on antidepressants and their clinical effects on inflammation/oxidation/mitochondrial dysfunction are often inconclusive or biased. Despite that, the combined evidence indicates that ATP depletion, oxidative stress, and inflammatory responses can activate apoptotic mechanisms that lead to neurodegeneration, a common feature in depressive patients. The data presented here demonstrate that MDD activates inflammation pathways through nuclear, cytosolic, and mitochondrial proteins, which can serve as a starting point for the screening of new drugs. However, the future challenge is to find effective new drugs that target inflammation and mitochondria, thus reducing cases of refractory patients. Finally, although the biochemistry mechanisms linked to the immune response in depression are gradually being elucidated, further research is needed to test these hypotheses in vivo.

## Figures and Tables

**Table 1:** Markers of oxidative stress and antioxidants related to drug-naive patients.

Study population	Outcome in DP	Reference
247 DP and 248 HC	↑ MDA	Islam et al. [139]
77 adult DP and 47 HC	↑ CP in DP at early stage; ↓ GPx activity at late stage	Diniz et al. [140]
19 adolescent DP and 8HC	↓ GSH	Freed et al. [141]
50 aged DP and 55 HC	↑ 8-OHdG	Lindqvist et al. [80]
50 DP and 50 HC	↑ MDA levels and ↓ SOD activity; no differences for CAT	Camkurt et al. [142]
21 DP and 40 HC	↑ CAT and SOD activities	Tsai and Huang [143]
332 symptomatic patients, 141 DP and 622 HC	↑ 8-OHdG	Black et al. [144]
49 adult DP and 49 HC	↑ TBARS and NO; ↓ SH; no differences for SOD	Kaufmann et al. [145]
60 DP and 40 HC	↑ MDA levels; ↓ SOD activity, nitrite and vitamin C levels	Bajpai et al. [146]
322 aged DP	↓ Vitamin C levels	Gariballa [147]
15 DP and 19 HC	↑ MDA and GR; ↓ SOD and GPx1	Rybka et al. [148]
82 adult DP and 94 HC	↑ CP	Magalhães et al. [149]
45 adult recurrent DP and 33 HC	↑ NO	Talarowska et al. [150]
70 aged DP and 35 HC	↓ GPx and GSH levels; ↑ GR and SOD activities; no differences for CAT	Kodydková et al. [151]
38 aged DP and 72 HC	↑ 8-OHdG	Kupper et al. [152]
35 DP and 35 HC	↓ CoQ10 levels	Maes et al. [153]

DP: depressive patients; HC: healthy controls; MDA: malondialdehyde; CP: carbonyl protein; GPx: glutathione peroxidase; SOD: superoxide dismutase; CAT: catalase; 8-OHdG: 8-hydroxydeguanosine; TBARS: thiobarbituric acid reactive species; NO: nitric oxide; SH: sulfhydryl; GR: glutathione reductase; GSH: glutathione; CoQ10: coenzyme Q10.

**Table 2:** Markers of oxidative stress and antioxidants related to antidepressant drugs (AD): preclinical and clinical studies.

Study model	Antidepressant drugs/dose	Outcome	Reference
Clinical	SSRIs, TCAs, other antidepressants	↓ 8-OHdG levels in antidepressant users	Black et al. [170]
	Sertraline, fluoxetine, citalopram, and escitalopram	↑ 8-OHdG levels in nonrespondent patients	Lindqvist et al. [80]
	Sertraline (25–100 mg)	↓ O_2_^·-^ and ^·^OH production; ↑ TRAP	Chang et al. [171]
	Escitalopram (20 mg)	↓ SOD, CAT, MDA, and NO levels	Cimen et al. [172]
	Venlafaxine, paroxetine, escitalopram, sertraline, citalopram, milnacipran, fluoxetine, tianeptine, and moclobemide	↓ SOD activity in red blood cells (24 weeks of treatment) (no difference exhibited after 6 and 12 weeks)	Kotan et al. [173]
	Fluoxetine (20 mg)	↓ MDA levels; ↓ SOD1, CAT, and GSHP-x activities; ↑ TAS	Galecki et al. [102]
	Fluoxetine (20 mg)	↑ ADA and SOD activities; ↓ NO and XO levels (no break down by medication group, reviewed meditations overall)	Herken et al. [174]
	Fluvoxamine (150 mg)		
	Sertraline (50 mg)		
	Citalopram (20 mg)		
	Fluoxetine and citalopram (20 mg)	↓ MDA levels; ↓ SOD activity; ↑ ascorbic acid levels	Khanzode et al. [175]
	Citalopram and fluoxetine (20 mg)	↓ MDA levels; ↓ GR, GPx, and SOD activities	Bilici et al. [176]
	Sertraline (50 mg)		
	Fluvoxamine (100 mg)		

Preclinical animal model	Escitalopram (5 mg/kg)	↓ ON in serum	Gammoh et al. [177]
	Ketamine (5 mg/kg)	Ketamine induced antioxidant or proantioxidant effects dependent on antidepressant classes or brain area	Réus et al. [178]
	Fluoxetine (1.25 mg/kg)		
	Lamotrigine (5 mg/kg)		
	Quetiapine (5 mg/kg)		
	Escitalopram (5 mg/kg)	↑ GSH in the brain	Matchkov et al. [179]
	Venlafaxine (5, 10, or 20 mg/kg)	↓ MDA and NO *plus* ↑ GSH and TAC levels and GST activity in the hippocampus; ↓ 8-OHdG levels in serum and the hippocampus	Abdel-Wahab and Salama [180]
	Fluoxetine (5 mg/kg)	Restored SOD, CAT, and GSH levels in peripheral blood leucocytes	Novío et al. [181]
	Imipramine (10, 20, or 30 mg/kg)	↑ SOD and CAT activities; ↓ MDA and carbonyl levels in the prefrontal cortex and hippocampus	Réus et al. [182]
	Fluoxetine (20 mg/kg)	Restored SOD, CAT, GST, and GR activities; ↑ GSH; ↓ MDA and carbonyl levels in the brain	Zafir et al. [183]
	Imipramine (10 mg/kg)		
	Venlafaxine (10 mg/kg)		
	Sertraline (5 or 10 mg/kg)	↑ Glutathione levels in the brain	P. Kumar and A. Kumar [184]

Preclinical cell culture	Clomipramine (15 *μ*M)	↓ NO production through attenuation of iNOS expression	Hwang et al. [185]
	Imipramine (10 *μ*M)		
	Desipramine (10^−5^, 10^−6^, or 10^−7^ M)	↓ mRNA levels of SOD and CAT after treatment (2.5 h) ↑ mRNA levels of SOD, GST, and GR after treatment (24 h)	Schmidt et al. [186]
	Imipramine (10^−5^, 10^−6^, or 10^−7^ M)		
	Maprotiline (10^−5^, 10^−6^, or 10^−7^ M)		
	Mirtazapine (10^−5^, 10^−6^, or 10^−7^ M)		
	Fluvoxamine (10 *μ*M)	Inhibition of NO production	Hashioka et al. [187]
	Imipramine (50 *μ*M)		
	Reboxetine (10 *μ*M)		
	Amitriptyline (50 or 100 *μ*mol/L)	Both agents attenuated cell death induced by H_2_O_2_; fluoxetine pretreatment ↑ SOD activity	Kolla et al. [188]
	Fluoxetine (50 *μ*mol/L)		

8-OHdG: 8-hydroxydeguanosine; ADA: adenosine deaminase; CAT: catalase; GPx: glutathione peroxidase; GR: glutathione reductase; GSH: glutathione; GST: glutathione S-transferase; H_2_O_2_: hydrogen peroxide; iNOS: nitric oxide synthase; MDA: malondialdehyde; NO: nitric oxide; PON: paraoxonase; SOD: superoxide dismutase; SOD1: copper-zinc superoxide dismutase; SSRI: selective serotonin reuptake inhibitor; TAC: total antioxidant capacity; TAS: total antioxidant status; TBARS: thiobarbituric acid reactive species; TCA: tricyclic or tetracyclic antidepressant; XO: xanthine oxidase; TRAP: radical-trapping antioxidant parameter.
